# *ADIPOQ* rs2241766 Gene Polymorphism and Predisposition to Diabetic Kidney Disease

**DOI:** 10.1155/2020/5158497

**Published:** 2020-06-28

**Authors:** Qiuxia Han, Wenjia Geng, Dong Zhang, Guangyan Cai, Hanyu Zhu

**Affiliations:** ^1^School of Medicine, Nankai University, Department of Nephrology, The First Medical Centre, Chinese PLA General Hospital, State Key Laboratory of Kidney Diseases, National Clinical Research Center of Kidney Diseases, China; ^2^Department of Nephrology, Guangdong Provincial Hospital of Chinese Medicine, Nephrology Institute of Guangdong Provincial Hospital of Chinese Medicine, The Second Affiliated Hospital of Guangzhou, University of Chinese Medicine, China; ^3^Department of Nephrology, The First Medical Centre, Chinese PLA General Hospital, Chinese PLA Institute of Nephrology, State Key Laboratory of Kidney Diseases, National Clinical Research Center of Kidney Diseases, Beijing Key Laboratory of Kidney Disease, China

## Abstract

**Background:**

This meta-analysis was performed to obtain a more comprehensive estimation of the role of the single nucleotide polymorphism (SNP) rs2241766 in the *ADIPOQ* gene in the occurrence of diabetic kidney disease (DKD).

**Methods:**

Relevant studies were identified from digital databases such as Embase, PubMed, Medline, Cochrane Library, Google Scholar, WanFang, and Chinese National Knowledge Infrastructure (CNKI). Odds ratios (ORs) with their corresponding 95% confidence intervals (95% CIs) were pooled by means of fixed- or random-effects models. Interstudy heterogeneity was examined using the Q test and *I*^2^ statistic, and sensitivity analysis was implemented to test the statistical stability of the overall estimates. Begg's funnel plot and Egger's test were applied to inspect potential publication bias among the included studies.

**Results:**

The overall ORs reflected a positive correlation between the *ADIPOQ* rs2241766 polymorphism and susceptibility to DKD in the GG vs. TT and GG vs. TT+TG comparisons (OR = 1.51, 95%CI = 1.16 − 1.95; OR = 1.43, 95%CI = 1.11 − 1.85). After stratification analyses by ethnicity and disease type, a similar trend was also revealed in the Caucasian and African subgroups as well as in the type 2 diabetes mellitus (T2DM) subgroup.

**Conclusion:**

The *ADIPOQ* rs2241766 polymorphism may be associated with an increased risk of DKD, especially in Caucasian and African populations as well as in T2DM patients.

## 1. Introduction

In diabetic patients, microvascular lesions and accelerated atherosclerosis tend to trigger complications leading to severe morbidity [1]. Diabetic kidney disease (DKD) is a representative microvascular complication of type 1 and type 2 diabetes mellitus (T1DM and T2DM), which can cause end-stage renal disease (ESRD) [2]. Reports show that approximately one-third of diabetic patients will eventually develop DKD, so it is of great significance to identify risk factors for DKD occurrence [3]. Patients suffering from DKD may ultimately require hemodialysis or even kidney transplantation, thus causing a serious economic healthcare burden [2]. DKD is a multifactorial disease occurring as a result of both environmental and hereditary factors [4, 5]. The ethnic disparity in DKD development may be attributed to an important role of genetic factors, and sex has also been demonstrated to influence the predisposition of diabetic patients to developing kidney diseases, with males having a relatively higher incidence rate [5].

One of the candidate genes for DKD is adiponectin (*ADIPOQ*), which has been indicated to be linked to susceptibility to cardiovascular disease, metabolic syndrome, and T2DM [6–8]. The *ADIPOQ* gene mapped to chromosome 3q27 consists of three exons and two introns [9]. This adipokine can exert anti-inflammatory and antiatherogenic effects and regulate glucose and lipid metabolism as well as insulin action [7]. The chromosomal region containing the *ADIPOQ* gene has been reported to be a cardiovascular risk factor as well [10]. Additionally, abnormal levels of serum adiponectin have already been shown to be correlated with T2DM, insulin resistance, obesity, cardiovascular diseases, and nephropathy [11]. Reportedly, the development of microalbuminuria in T1DM cases may be predicted by high adiponectin levels [5, 12]. Several single nucleotide polymorphisms (SNPs) have been identified in the *ADIPOQ* gene, and their associations with the risk of DKD in T1DM and T2DM patients have been investigated in diverse populations in many case-control studies, but the results remain inconclusive.

In the present meta-analysis, we selected one commonly studied SNP, rs2241766, in exon 2 of the *ADIPOQ* gene to clarify its effects on DKD occurrence based on previously published case-control studies on this topic.

## 2. Materials and Methods

### 2.1. Search Strategy

A comprehensive literature search was conducted in electronic databases, including Embase, PubMed, Medline, Cochrane Library, Google Scholar, Wanfang, and Chinese National Knowledge Infrastructure (CNKI), using different combinations of the following keywords: “diabetic kidney disease” or “DKD” or “diabetic nephropathy” or “DN” or “diabetic renal disease” or “DRD” or “diabetic end-stage renal disease” or “diabetic ESRD” or “diabetic renal dysfunction” or “diabetic kidney failure” or “diabetic microalbuminuria” or “diabetic albuminuria” or “diabetic proteinuria” or “diabetic glomerulosclerosis” or “Kimmelstiel-Wilson Syndrome” or “Kimmelstiel-Wilson Disease” or “diabetic complications”, and “adiponectin” or “*ADIPOQ*”, and “polymorphism” or “variant” or “mutant” or “single nucleotide polymorphism” or “SNP” or “genotype” or “allele”. Further relevant articles were identified by reviewing the reference lists of the included articles.

### 2.2. Selection Criteria

The following inclusion criteria were set for the present meta-analysis: (1) studies including both case and control subjects; (2) studies evaluating the correlation between the *ADIPOQ* rs2241766 polymorphism and susceptibility to DKD; (3) studies providing sufficient information such as genotype frequency for evaluation of odds ratios (ORs) and 95% confidence intervals (95% CIs); and (4) full-text articles of studies with human subjects. Studies meeting any one of the following criteria were considered ineligible for the present meta-analysis: (1) conference abstracts, comments, reviews, case reports, or editorials; (2) insufficient data for OR calculation; (3) no control group; and (4) animal studies.

### 2.3. Quality Assessment

We evaluated the quality of all included studies using the Newcastle-Ottawa Scale (NOS). The NOS is composed of 3 aspects: selection, comparability, and exposure, with a total score of 9. According to the final score, the studies could be categorized into high quality (score more than 6), medium quality (score between 4 and 6), and low quality (score less than 4).

### 2.4. Data Extraction

Data extraction was conducted by two reviewers independently. Conflicting opinions were resolved through discussion to reach a final consensus. The items extracted from each eligible study included the first author's name, publication year, region, ethnicity, disease type, total cases and controls, genotype and/or allele frequencies in case and control groups, genotyping method, and evidence of Hardy-Weinberg equilibrium (HWE) in controls.

### 2.5. Statistical Analysis

STATA software (version 12.0) was used to conduct all data syntheses in this meta-analysis. The strength of the relationship between the *ADIPOQ* rs2241766 polymorphism and DKD susceptibility was determined by calculating pooled ORs and 95% CIs. The interstudy heterogeneity assumption was examined by means of *χ*^2^-based Q-statistic and *I*^2^ tests. If heterogeneity was significant (*p* < 0.05 of the Q test or *I*^2^ > 50%), the summarized OR estimates were calculated utilizing the random-effects model (DerSimonian and Laird method); otherwise, the fixed-effects model (Mantel-Haenszel method) was applied. Additionally, when the heterogeneity between studies is statistically significant, we would use meta-regression analysis to identify potential sources of such heterogeneity. Begg's funnel plots and Egger's linear regression test were used to evaluate possible publication bias among the included studies. The stability of the combined results was examined by performing a sensitivity analysis, in which each of the included studies was sequentially deleted, and then summary ORs were recalculated to observe alterations between the original and reobtained ORs. The statistical significance of all tests was denoted at *p* < 0.05.

## 3. Results

### 3.1. Characteristics of Studies

**Figure 1:**
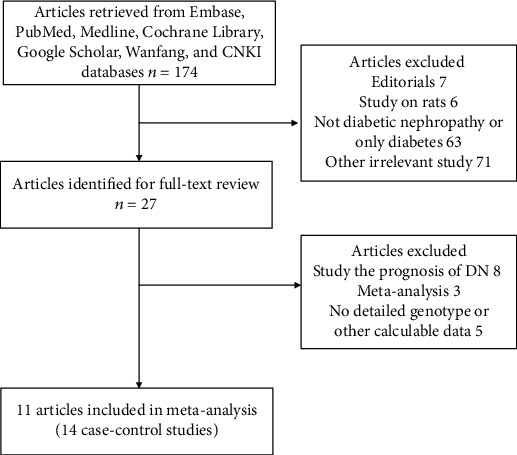
Flowchart illustrating the process of study identification, inclusion, and exclusion.

The publication search is described in Figure 1. Initially, 174 articles were identified from the database search. During further review, 163 articles were deleted due to being editorials (7), being conducted on rats (6), not being about DKD or merely being about diabetes (63), being obviously irrelevant (71), being related to the prognosis of DKD (8), being a meta-analysis (3), and having no detailed data about genotype and allele frequencies (5). Finally, 14 case-control studies with 3343 cases and 7859 controls were incorporated into the present meta-analysis [13–23]. All of the controls in our meta-analysis were diabetic patients without nephropathy. Additionally, most of the included studies were of high quality (NOS score of more than 6).  Table 1 summarizes the general characteristics of the incorporated studies.

### 3.2. Meta-Analysis Results

**Figure 2:**
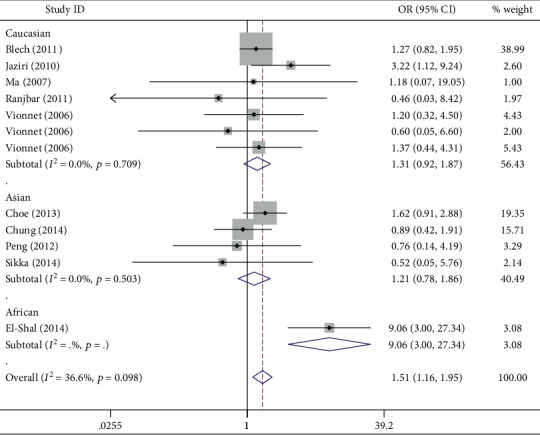
Forest plot for the association between the *ADIPOQ* rs2241766 polymorphism and DKD risk in the GG vs. TT comparison. The squares and horizontal lines correspond to the study-specific OR and 95% CI. The area of the squares reflects the weight (inverse of the variance). The diamond represents the summary OR and 95% CI.

The relationship between the *ADIPOQ* rs2241766 polymorphism and DKD susceptibility is illustrated in Table 2. The combined results demonstrated that the *ADIPOQ* rs2241766 polymorphism increased susceptibility to DKD in two genetic comparisons of GG vs. TT (Figure 2) and GG vs. TT+TG (OR = 1.51, 95%CI = 1.16 − 1.95; OR = 1.43, 95%CI = 1.11 − 1.85). A risk-increasing effect of the polymorphism was also shown in Caucasian (GG+TG vs. TT: OR = 1.27, 95%CI = 1.01 − 1.60; allele G vs. allele T: OR = 1.12, 95%CI = 1.01 − 1.25)), African (GG vs. TT: OR = 9.06, 95%CI = 3.00 − 27.34 (Figure 2); GG+TG vs. TT: OR = 4.80, 95%CI = 2.86 − 8.03; GG vs. TT+TG: OR = 4.34, 95%CI = 1.48 − 12.68; allele G vs. allele T: OR = 3.16, 95%CI = 2.12 − 4.71; TG vs. TT: OR = 4.26, 95%CI = 2.50 − 7.28), and T2DM (GG vs. TT: OR = 1.79, 95%CI = 1.26 − 2.55; GG vs. TT+TG: OR = 1.68, 95%CI = 1.18 − 2.39) groups after subgroup analyses were conducted by ethnicity and disease type.

### 3.3. Heterogeneity Test

The statistical Q test and *I*^2^ statistic revealed significant heterogeneity in the GG+TG vs. TT, G vs. T and TG vs. TT comparisons, so the random-effects model was chosen for calculating ORs in these cases, while the fixed-effects model was adopted for the other two genetic comparisons in which heterogeneity was negligible.

In the three comparisons in which significant heterogeneity was revealed, meta-regression analysis was conducted, and the results demonstrated that differences in ethnic origin could explain the vast majority or even all of the sources of the significant heterogeneity.

### 3.4. Sensitivity Analysis

**Figure 3:**
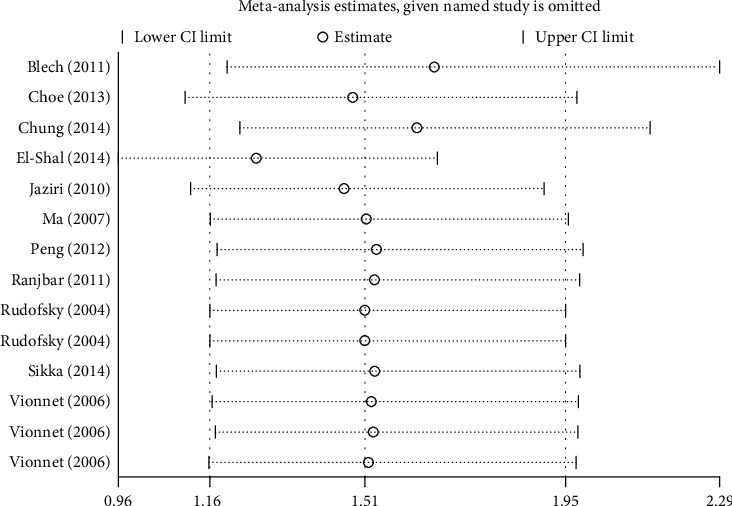
Sensitivity analysis for testing the stability of the overall estimate in the GG vs. TT comparison.

In the sensitivity analysis, recalculated ORs after removing any single eligible study showed that no material alterations were detected from the original ORs (Figure 3), implying that our results were reliable and robust.

### 3.5. Publication Bias

**Figure 4:**
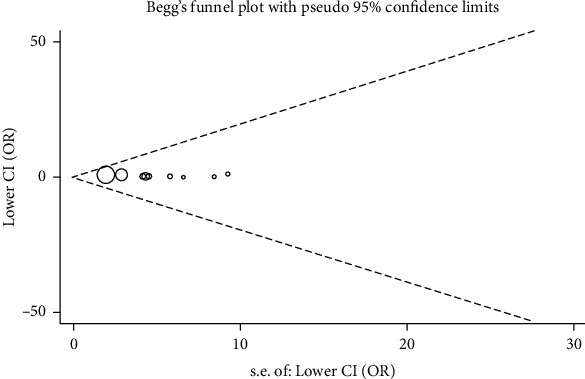
Begg's funnel plot for publication bias in the GG vs. TT comparison. Each point represents a separate study for the indicated association.

Potential publication bias among the included studies was assessed through the visual inspection of Begg's funnel plots accompanied by statistical results from Egger's test. The symmetrical shape of the funnel plots (Figure 4) and *p* value of Egger's linear regression test (*p* = 0.451) indicated the absence of significant publication bias.

## 4. Discussion

According to existing evidence, high adiponectin levels in T1DM and T2DM patients may be related to the pathogenesis of diabetic nephropathy [24–29]. The adiponectin protein encoded by the *ADIPOQ* gene can prevent vascular remodeling by inhibiting the proliferation and migration of smooth muscle cells and reduce TNF-*α* production to modulate the inflammatory response of endothelial cells [30, 31]. In addition, adiponectin can protect the vasculature through its pleiotropic actions on endothelial progenitor cells, endothelial cells, macrophages, and smooth muscle cells [32]. Moreover, adiponectin may also prevent the injury and dysfunction of endothelial cells due to its protective effects [32]. Genetic polymorphisms in the *ADIPOQ* gene can affect adiponectin levels, and their contribution to the occurrence of DKD, a common microvascular complication, has been frequently discussed, but conflicting results have been yielded.

We therefore performed the present meta-analysis including 3346 cases and 7859 controls to obtain better insight into the relationship between the *ADIPOQ* rs2241766 polymorphism and DKD risk. After data synthesis, we observed a risk-increasing effect of the *ADIPOQ* rs2241766 polymorphism on DKD susceptibility in the GG vs. TT and GG vs. TT+TG models. The subgroup analyses based on ethnicity and disease type also revealed this positive correlation between the SNP and disease risk in Caucasian, African, and T2DM groups.

The results of previous studies regarding the effects of the *ADIPOQ* rs2241766 polymorphism on susceptibility to DKD remain inconclusive. In a study among patients with T2DM, the GG genotype of the rs2241766 polymorphism was found to be significantly associated with the risk of DKD after adjusting for confounding factors [16]. In a study among Taiwanese individuals by Chung et al., the SNP was also observed to participate in the progression of DKD in TT vs. GT+GG, GG/GT/TT, and allele T vs. allele G models among male subjects [15]. Another study among an Egyptian population also suggested a similar correlation of the SNP with DKD susceptibility [14]. In contrast, Ma et al. found no independent role of rs2241766 in nephropathy development among Swedish Caucasians [22], and three other studies by Sikka et al., Peng et al., and Rudofsky et al. obtained similar results [13, 17, 23].

There are several possible reasons for the above controversy. First, subjects recruited by the above studies belonged to different ethnic groups. Second, confounding exogenous factors such as age, sex, and lifestyle factors were not adjusted in all studies. Third, the limited number of study participants might reduce the authoritativeness of some study results. Of course, some meta-analyses related to our studied topic have already been performed, such as the one by Lin et al. [33] and one by Cai et al. [34]. However, the meta-analysis by Lin and colleagues only included 7 articles describing 9 independent studies on our studied polymorphism, while we included 14 eligible studies from 11 papers. The meta-analysis by Cai and colleagues was only related to DKD in type 2 diabetes patients.

Compared to the above studies, our meta-analysis has many advantages, such as a relatively larger sample size. However, some limitations of the present study should also be mentioned. To begin with, unpublished studies with null results were not included in this meta-analysis, thus possibly introducing certain publication bias, though this bias was not significant. Next, the number of studies for stratification analyses was relatively small, thus affecting the comprehensiveness of the conclusions. Finally, possible interactions of our studied SNP with other relevant factors were not analyzed owing to limited information.

## 5. Conclusion

Overall, the present meta-analysis indicated that the *ADIPOQ* rs2241766 polymorphism might be related to an increased risk of DKD occurrence, which was more evident in Caucasian and African populations as well as among T2DM patients. In view of the abovementioned limitations, these results need to be further verified in the future by studies with larger sample sizes.

## Figures and Tables

**Table 1:** Main characteristics of the included studies in the present meta-analysis.

First author (year)	Country	Ethnicity	Control source	Type	Sample size	Case genotype			Control genotype			Genotyping method	NOS score	P_(HWE)_
					Case/control	TT	TG	GG	TT	TG	GG			
Blech (2011)	Israel	Caucasian	Diabetic	T2DM or T1DM	852/1473	532	283	37	966	454	53	PCR-RFLP	7	0.999
Choe (2013)	Korea	Asian	Diabetic	T2DM	245/448	124	95	26	216	204	28	SNaPShot	7	0.083
Chung (2014)	China	Asian	Diabetic	T2DM	144/422	77	57	10	206	186	30	Multiplex PCR	8	0.386
El-Shal (2014)	Egypt	African	Diabetic	T2DM	196/100	53	113	30	64	32	4	PCR-RFLP	7	1
Jaziri (2010)	France	Caucasian	Diabetic	T2DM	75/3011	46	25	4	2223	728	60	PCR-MB	8	0.999
Ma (2007)	Sweden	Caucasian	Diabetic	T1DM	196/236	180	15	1	213	22	1	PCR-DASH	7	0.871
Peng (2012)	China	Asian	Diabetic	T2DM	42/40	25	14	3	19	18	3	DS	7	0.903
Ranjbar (2011)	Iran	Caucasian	Diabetic	T2DM	28/205	20	8	0	142	56	7	PCR-RFLP	7	0.880
Rudofsky (2004)	Germany	Caucasian	Diabetic	T1DM	73/166	47	26		147	19		PCR-RFLP	8	N/A
Rudofsky (2004)	Germany	Caucasian	Diabetic	T2DM	174/283	137	37		239	44		PCR-RFLP	8	N/A
Sikka (2014)	India	Asian	Diabetic	T2DM	145/152	124	20	1	128	22	2	PCR-RFLP	6	0.654
Vionnet (2006)	Denmark	Caucasian	Diabetic	T1DM	489/463	393	91	5	377	82	4	Ampli-Fluor	7	0.981
Vionnet (2006)	Finland	Caucasian	Diabetic	T1DM	387/469	349	37	1	416	51	2	Ampli-Fluor	7	0.949
Vionnet (2006)	France	Caucasian	Diabetic	T1DM	300/391	221	73	6	303	82	6	Ampli-Fluor	7	0.986

PCR: polymerase chain reaction; PCR-RFLP: PCR-restriction fragment length polymorphism; PCR-MB: PCR-molecular beacon; PCR-DASH: PCR-dynamic allele-specific hybridization; DS: Direct sequencing.

**Table 2:** Meta-analysis of the association between the *ADIPOQ* rs2241766 polymorphism and DKD risk.

Model	Ethnicity			Type			Total	No. of studies and participants
	Caucasian	Asian	African	T1DM	T2DM	T2DM or T1DM		
GG vs. TT								
OR (95% CI)	1.31 (0.92, 1.87)	1.21 (0.78, 1.86)	9.06 (3.00, 27.34)	1.18 (0.54, 2.56)	1.79 (1.26, 2.55)	1.27 (0.82, 1.95)	1.51 (1.16, 1.95)	12 (324/7417)
Ph	0.709	0.503	/	0.945	0.016	/	0.098	
*I*^2^ test	0.0%	0.0%	/	0.0%	61.6%	/	36.6%	
GG + TG vs. TT								
OR (95% CI)	1.27 (1.01, 1.60)	0.86 (0.69, 1.07)	4.80 (2.86, 8.03)	0.99 (0.80, 1.24)	1.25 (0.81, 1.93)	1.15 (0.96, 1.37)	1.12 (0.90, 1.40)	14 (3218/7987)
Ph	0.006	0.864	/	0.312	0.000	/	0.000	
*I*^2^ test	62.9%	0.0%	/	16.1%	83.0%	/	72.9%	
GG vs. TT+TG								
OR (95% CI)	1.26 (0.88, 1.79)	1.34 (0.88, 2.03)	4.34 (1.48, 12.68)	1.15 (0.53, 2.50)	1.68 (1.18, 2.39)	1.22 (0.79, 1.87)	1.43 (1.11, 1.85)	12 (324/10185)
Ph	0.800	0.483	/	0.955	0.229	/	0.537	
*I*^2^ test	0.0%	0.0%	/	0.0%	26.1%	/	0.0%	
G vs. T								
OR (95% CI)	1.12 (1.01, 1.25)	0.95 (0.80, 1.14)	3.16 (2.12, 4.71)	1.05 (0.87, 1.26)	1.19 (0.81, 1.75)	1.13 (0.97, 1.31)	1.13 (0.93, 1.38)	12 (3416/17602)
Ph	0.245	0.690	/	0.540	0.000	/	0.000	
*I*^2^ test	24.1%	0.0%	/	0.0%	82.8%	/	71.4%	
TG vs. TT								
OR (95% CI)	1.12 (0.98, 1.27)	0.81 (0.65, 1.02)	4.26 (2.50, 7.28)	1.04 (0.85, 1.27)	1.18 (0.72, 1.91)	1.13 (0.94, 1.36)	1.11 (0.88, 1.40)	12 (1937/5273)
Ph	0.547	0.885	/	0.580	0.000	/	0.000	
*I*^2^ test	0.0%	0.0%	/	0.0%	82.7%	/	70.3%	

Note: Ph: *p* value of heterogeneity; if Ph is >0.05, a fixed-effect model was used to calculate OR and 95% CI; otherwise, a random-effect model was used.
